# Balance Rehabilitation Unit (BRU^TM^) posturography in Menière's disease

**DOI:** 10.1590/S1808-86942010000500013

**Published:** 2015-10-22

**Authors:** Flavia Salvaterra Cusin, Maurício Malavasi Ganança, Fernando Freitas Ganança, Cristina Freitas Ganança, Heloisa Helena Caovilla

**Affiliations:** 1 MSc in Sciences - Graduate Program in Human Communication Disorders - Federal University of São Paulo - Paulista School of Medicine (UNIFESP/EPM). Speech and Hearing Therapist; 2 Full Professor of Otorhinolaryngology - Federal University of São Paulo - Paulista School of Medicine (UNIFESP/EPM). Professor - MSc Program in Body Balance Rehab and Social Inclusion - Universidade Bandeirante de São Paulo (UNIBAN); 3 Adjunct Professor of Otology and Neurotology - Federal University of São Paulo - Paulista School of Medicine (UNIFESP/EPM). Professor - MSc Program in Body Balance Rehab and Social Inclusion - Universidade Bandeirante de São Paulo (UNIBAN); 4 PhD in Sciences - Graduate Program in Human Communication Disorders - Federal University of São Paulo - Paulista School of Medicine (UNIFESP/EPM); 5 Associate Professor - Otology and Neurotology Program - Federal University of São Paulo - Paulista School of Medicine (UNIFESP/EPM). Universidade Federal de São Paulo - Escola Paulista de Medicina (UNIFESP-EPM)

**Keywords:** vestibular function tests, meniere's disease, ear, dizziness, inner.

## Abstract

**Abstract:**

Posturography has been used in the evaluation of patients with vestibular disorders.

**Aim:**

To evaluate balance control with the Balance Rehabilitation Unit (BRU^TM^) posturography in patients with Menière's disease.

**Study design:**

Prospective case-control.

**Material and Method:**

30 patients diagnosed with Menière's disease and a control group consisting of 40 healthy matching individuals in relation to age and gender, were submitted to a balance function evaluation by means of a Balance Rehabilitation Unit (BRU^TM^) posturography.

**Results:**

Comparing patients with Menière's disease and the control group, we found significant differences between the values of the sway speed in the static force plate, down optokinetic stimulation (p=0.038) and horizontal visual vestibular interaction (p=0.049); and of the ellipse area in the static force plate, eyes closed (p=0.001); left optokinetic stimulation (p=0.007); down optokinetic stimulation (p=0.003); horizontal visual vestibular interaction (p=0.003); and vertical visual vestibular interaction (p=0.028).

**Conclusion:**

The postural control assessment with the Balance Rehabilitation Unit (BRU^TM^) posturography enables the identification of sway speed and ellipse area abnormalities in patients with Menière's disease.

## INTRODUCTION

The symptoms triad of tinnitus, hearing loss and vertigo, in paroxysmal episodes, without central nervous system involvement was described by Prosper Ménière in 1861 to the Imperial Academy of Medicine of Paris. For the first time it was suggested that the auditory system could be suddenly affected with tinnitus and hearing loss; the inner ear would be the site of involvement; and vertigo, dizziness and unbalance, followed by nausea, vomit and syncope, could be explained without the involvement of the central nervous system^1^. These manifestations were called Ménière's Disease^2^ and after histopathology descriptions, Ménière's Disease was finally recognized as the clinical expression of an idiopathic syndrome of endolymphatic hydrops^3^.

The final confirmation of pathophysiological change, which characterizes the disease, can only be proven by means of a pathology exam of the temporal bones (post mortem) and, therefore, diagnosis must be based on well defined clinical criteria. The presence of endolymphatic hydrops can be inferred from the occurrence of spontaneous and recurrent episodes of vertigo, lasting for at least 20 minutes, followed by nausea and/or vomit, without loss of conscience, horizontal-rotational nystagmus during the crisis, associated with hearing loss, ear fullness and tinnitus on the affected side^4^.

Ménière's Disease is one of the most prevalent vestibular disorders, for each 100 thousand individuals, there is a prevalence of 157 cases in the United Kingdom, 46 cases in Sweden, 7.5 cases in France and 15 cases in the USA^5^. There is no distribution difference between the genders and it usually manifests on the fourth decade of life^6^.

It is fundamental that patients with Ménière's Disease be submitted to a broad neurotological evaluation in order to characterize the changes associated with the complaints of dizziness and unbalance. As part of this evaluation, the static posturography module of the Balance Rehabilitation Unit (BRU^TM^) provides information on the patient's pressure center position under ten sensorial situations by means of quantitative indicators, stability limit area, elliptical area and oscillation velocity^7^.

Posturography is an efficient tool to assess static body balance in patients with vestibular changes^8^; such method checks the vestibular-spinal reflex by means of recording and analyzing postural oscillation and it may identify impairments in the integration of visual, vestibular and proprioceptive afferent stimuli by means of exposing the patient to different sensorial situations^9^, ^10^, ^11^.

Different types of static posturography were used in order to assess the influence of the vestibular-spinal reflex on the maintenance of body balance in patients with Ménière's Disease^8^, ^9^, ^10^, ^11^, ^12^, ^13^. These studies identified an abnormal posture oscillation in 46.7% of the cases in at least of the sensorial situations evaluated, open and shut eyes on the platform, and the worst results were obtained with the eyes shut^8^, ^9^; in 52.8% of the cases, similarly on the six sensorial situations analyzed, on the platform with the eyes open, shut and the head in retroflexion with eyes shut and, on a foam pillow with the eyes open, shut and head in retroflexion with the eyes shut^10^; in 50% of the cases with open eyes on the platform and in 24.6% on the other three sensorial situations evaluated, eyes shut on the platform and eyes open and shut on the pillow^11^; in 42% of the patients with the eyes open and in 45% with the eyes shut, regardless of disease duration, the number of patients with normal values with their eyes open reduced with the progress of the affection and the oscillation was greater in the cases of moderate hearing loss when compared to mild hearing loss^12^; in 53.3% of the cases, in at least one of the four sensorial situations evaluated, open and shut eyes on a platform and open and shut eyes on a foam pillow, and the worst results were seen on the foam pillow^13^.

We did not find references as to the use of Balance Rehabilitation Unit (BRU^TM^) posturography in order to assess the body balance of patients with overt Ménière's Disease, and this was the reason as to why we did the present investigation.

The goal of this study was to assess body balance using the static Balance Rehabilitation Unit (BRU^TM^) posturography in patients diagnosed with overt Ménière's Disease.

## MATERIALS AND METHODS

This study was previously approved by the Ethics in Research Committee of the University (protocol number 01804/07). A free and informed consent was obtained from all the patients before the onset of the investigation.

In this controlled cross-sectional experimental study, the sample was made up of a group of 30 adult male and female patients with a diagnostic hypothesis of overt Ménière's Disease and a control group made up of 40 healthy homogeneous individuals insofar as age and gender are concerned.

The patient inclusion criteria in the experimental group were: diagnosis hypothesis of overt Ménière's Disease, characterized by two or more episodes of vertigo lasting 20 or more minutes, documented hearing loss in at least one occasion by means of audiometry, tinnitus or a feeling of pressure on the affected ear4, not having a vertigo spell and not having undergone previous treatment or not having been treated at all in the past three months.

We excluded those patients unable to understand and follow simple verbal commands; making it impossible for them to stand up alone, those with severe visual impairment or not compensated through the use of corrective lenses; those with orthopedic disorders which resulted in movement limitation or those with prosthesis in both lower limbs; those with neurological and/or psychiatric disorders; those who drank alcoholic beverages within 24 hours before the assessment; those using medication which act on the central nervous system or vestibular system, and those who have been through body balance rehabilitation in the past six months.

The patients in the experimental group were submitted to a neurotological evaluation in order to characterize their clinical picture, considering all the phases of the disease. This evaluation was made up of an interview in order to collect data about dizziness, hearing loss, tinnitus, ear fullness and associated symptoms; visual inspection of the external acoustic meatus; threshold tonal audiometry; speech recognition threshold; speech recognition percentage index; tympanometry; acoustic reflex investigation; electrocochleography; Brazilian Version^15^ of the Dizziness Handicap Inventory^14^; dizziness analogue scale^16^; positional and positioning nystagmus; computerized vectoreletronystagmography and Balance Rehabilitation Unit (BRU^TM^)^17^ posturography. The patients from the control group were submitted to a medical interview in order to characterize the lack of neurotological symptoms; and, Balance Rehabilitation Unit (BRU^TM^) posturography.

The Balance Rehabilitation Unit (BRU^TM^)^17^ posturography was carried out in a silent and dim room of about six square meters. The equipment included a computer with the evaluation software, safety metal structure, protection support system with harnesses and a safety belt, a force platform, virtual reality goggles, accelerometer and a foam pillow^15^.

The evaluation was carried out with the patient standing up with the arms extended along the body and when required, the patients were allowed to wear corrective lenses. The patient stood up on the platform, bare feet, with the right and left internal malleolus on the extremities of the intermalleolar line and the first two toes spread at 10 degrees from the middle line. The middle point of the intermalleolar line was used as the center of the standard limit of the stability circle.

In order to establish the stability limit, the patient was instructed to shift his body in an antero-posterior and lateral directions using the ankle strategy, without moving the feet or using trunk strategies.

In order to assess the ten sensorial situations, the patient was instructed to keep still for 60 seconds:

1) orthostatic position on a firm ground, with the eyes open;
2) orthostatic position on a firm ground, with the eyes shut;
3) orthostatic position on the foam pillow, with the eyes shut;
4) orthostatic position on the firm ground, with saccadic stimulation;
5) orthostatic position on firm ground, with horizontal optokinetic stimulation from left to right;
6) orthostatic position on firm ground, with horizontal optokinetic stimulation right to left;
7) orthostatic position on firm ground, with vertical optokinetic from top to bottom;
8) orthostatic position on firm ground, with vertical optokinetic stimulation from bottom up;
9) orthostatic position on firm ground, with horizontal optokinetic stimulation associated with slow and uniform head rotation movements;
10) orthostatic position on firm ground with vertical optokinetic stimulation associated with slow and uniform flexion and extension head movements.

A medium density foam pillow was used in the assessment of the third sensorial situation. The virtual reality goggles were used in the evaluations of the fourth to the tenth situations. The software generated reports with information on the stability limit area, the 95% confidence elliptical area and the oscillatory speed on the ten sensorial situations.

We carried out a descriptive statistical analysis in order to characterize the sample. The Chi-Square test (c2) was used in order to test the homogeneity between gender ratios between the groups. The T-Student test was used in the comparative analysis of the groups and age, and also that between the groups and the stability limit. We used the parametric test because of the symmetry and adherence to the normal distribution of the Kolmogorov-Smirnov normality test. The T-Student Test was used in order to look for differences between the oscillatory velocity mean values and that of the elliptical area in the BRUTM situations between the Ménière's Disease group and the control group. The normality assumption was rejected by the Kolmogorov-Smirnov test and, therefore, these variables were transformed by means of the logarithmic function. In the tests in which there were significant differences between the groups we calculated the test's power. The values found varied between 60% and 90%, showing that the sample size was sufficient in order to obtain approximately 80% of the power. The analyses were carried out using the SPSS 10.0 for Windows (Statistical Package for Social Sciences, version 10.0, 1999) software; the level of significance used for the statistical tests was 5% (a = 0.05)^18^.

## RESULTS

We assessed 40 individuals in the control group and 30 patients with Ménière's Disease in the intercritical period of the disorder. The control group was made up of nine male individuals (22.5%) and 31 females (77.5%). The group of patients with Ménière was made up of eight men (26.7%) and 22 women (73.3%). We did not find statistically significant differences between the groups as far as gender goes (p=0.687).

Insofar as age is concerned, the control group had mean age of 45.55 ± 12.36 years and the experimental group had mean age of 45.67 ± 13.01 years. We did not notice any significant difference between the groups as far as mean age is concerned (p=0.970).

As to dizziness, we noticed that in 18 cases (60.0%) dizziness was rotational; in seven (23.3%) it was rotational and non-rotational and in five (16.7%) it was non-rotational; in 16 cases (53.3%) it lasted for days; in nine (30.0%) it lasted for hours; and in five (16.7%) it lasted minutes; in 13 cases (43.3%) it was weekly; in eight (26.7%) it was daily; in eight (26.7%) sporadic and in one (3.3%) monthly; in 13 cases (43.3%) it had started over five years ago; in seven (23.3%) from 1 to 2 years; in five (16.7%) from 3 to 4 years; in four (13.3%) from three to six months; and in one (3.3%) in 8 months.

The mean score found in the dizziness analogue scale was 7.43 points (standard deviation = 1.99).

The mean score found in the life quality questionnaire was 59.80 points (standard deviation = 23.33) for the total score of 17.80 points (standard deviation = 6.57) for physical aspect, 20.53 points (standard deviation = 9.87) for the emotional aspect; and 21.47 points (standard deviation = 10.07) for the functional aspect.

Hearing loss, ear fullness and tinnitus were reported by all 30 patients in one or both ears. Nausea, vomit, paleness and tachycardia were reported by 27 cases (90.0%), hypersensitivity to sound by 25 patients (83.3%), memory disorders and concentration disorders were reported by 19 cases (63.3%), headache by 17 (56.7%) and stress by four (13.3%).

We found 20 cases of unilateral sensorineural hearing loss (66.7%); mild in 13, moderate in 6 and profound in one patient. Bilateral sensorineural hearing loss was found in 10 cases (33.3%); mild in five, moderate in three - moderate in one ear and mild in the other and one case of moderate in one ear and severe in the other.

Upon electrocochleography, we found a rela- tionship between the summation potential and the action potential above 30% in 30 cases; unilateral in 27 (90.0%) and bilateral in three cases (10.0%).

Upon vestibulometry, 13 patients (43.3%) had normal results and 17 (56.7%) had peripheral changes, of which six had vestibular hyporeflexia on the caloric test.

Upon Balance Rehabilitation Unit (BRU^TM^) posturography, there were no statistically significant differences (p=0.635) between the values of the stability limit area (cm2) of the control group (mean = 184.60; standard deviation = 48.46; median = 188.50; variation = 91-277) and the values from the group with Ménière's Disease (mean =181.43; standard deviation =59.76; median = 174.00; variation = 70-292).

Table 1 shows the descriptive values and comparative analysis of the oscillatory velocity (cm/s) in the groups control and Ménière's Disease. The mean values of oscillatory speed in the experimental group were higher than those in the control group under all situations assessed, with significant differences in the firm surface situation and downwards optokinetic stimulation (p=0.038) and that of the firm surface and horizontal visual-vestibular interaction (p=0.049).

**Table 1:** Descriptive values and comparative analysis of the oscillatory velocity (cm/s) in the Balance Rehabilitation Unit (BRU™) of 40 individuals from the control group and 30 patients in the intercritical period of overt Ménière's disease.

Oscillatory velocity (cm/s) of the BRU™	Groups	Mean	Standard deviation	Minimal value	Maximum value	Median	p-value*
FS/Open eyes/No stimulus	MD Control	0,80 0,73	0,45 0,27	0,47 0,39	2,88 1,83	0,66 0,66	0,551
FS/Closed eyes	MD Control	1,13 0,90	0,63 0,27	0,54 0,54	3,75 1,85	0,95 0,87	0,079
Foam/eyes closed	MD Control	2,49 2,44	0,96 0,94	1,23 0,71	5,35 5,48	2,34 2,23	0,827
FS/Saccadic	MD Control	1,01 0,98	0,52 0,43	0,43 0,51	3,26 2,94	0,84 0,86	0,856
FS/Bars/Optokinetic to the right	MD Control	1,00 0,88	0,32 0,26	0,60 0,42	1,73 1,51	0,99 0,805	0,083
FS/Bars/Optokinetic to the left	MD Control	1,07 0,88	0,53 0,29	0,52 0,44	3,07 1,78	0,97 0,89	0,076
FS/Bars/Optokinetic downwards	MD Control	1,08 0,89	0,45 0,28	0,51 0,48	2,46 1,60	0,99 0,86	0,038
FS/Bars/Optokinetic upwards	MD Control	1,08 0,91	0,46 0,32	0,46 0,44	2,12 2,00	0,98 0,84	0,105
FS/Visual-vestibular interaction/Horizontal direction	MD Control	1,40 1,18	0,58 0,44	0,75 0,20	3,87 2,45	1,27 1,07	0,049
FS/Visual-Vestibular interaction/Vertical direction	MD Control	1,47 1,30	0,60 0,41	0,74 0,61	3,54 2,26	1,35 1,20	0,151

Legend:

MD: Ménière's Disease

FS: Firm Surface

* T-Student test Significance level α = 0.05

Table 2 shows the descriptive values and the comparative analysis of the elliptical area (cm^2^) in the control and Ménière groups. The mean values of the elliptical area of the experimental group were larger than those in the control group under all situations analyzed, with significant differences considering the firm surface and eyes shut (p=0.001); of firm surface and left side optokinetic stimulation (p=0.007); firm surface and downward optokinetic stimulation (p=0.003); firm surface and horizontal visual-vestibular interaction (p=0.003), and firm surface and vertical visual-vestibular interaction (p=0.028).

**Table 2:** Critical values and comparative analysis of the elliptical area (cm^2^) of the Balance Rehabilitation Unit (BRU^TM^) of 40 individuals from the control group and 30 patients in the intercritical period of the overt Ménière's disease.

Elliptical area (cm^2^) of BRU^TM^	Groups	Mean	Standard deviation	Minimum value	Maximum value	Median	p-value*
FS/Open eyes/No Stimulus	MD Control	2,83 1,68	2,25 0,93	0,19 0,46	7,72 3,61	1,88 1,52	0,095
FS/Eyes shut	MD Control	4,49 1,80	5,50 1,15	0,80 0,32	24,35 4,61	2,12 1,55	0,001
Foam/Eyes shut	MD Control	9,70 7,62	7,89 4,33	3,48 2,44	44,74 19,92	7,35 6,39	0,137
FS/Saccadic	MD Control	1,95 1,46	1,43 1,15	0,34 0,23	6,58 5,31	1,39 1,02	0,071
FS/Bars/Optokinetic to the right	MD Control	2,62 1,57	2,89 1,01	0,57 0,36	12,87 4,70	1,50 1,23	0,080
FS/Bars/Optokinetic to the left	MD Control	3,50 1,43	4,56 0,99	0,42 0,30	20,41 4,15	1,63 1,11	0,007
FS/Bars/Optokinetic downwards	MD Control	3,26 1,58	3,24 1,47	0,47 0,37	10,94 7,07	1,82 1,11	0,003
FS/Bars/Optokinetic upwards	MD Control	3,90 1,82	5,13 1,64	0,33 0,41	20,68 6,95	1,52 1,05	0,080
FS/Visual-Vestibular Interaction/Horizontal Direction	MD Control	4,87 2,31	5,11 1,82	0,38 0,06	18,95 10,31	2,64 1,82	0,003
FS/Visual-Vestibular Interaction/Vertical Direction	MD Control	4,30 2,41	4,13 1,58	0,61 0,29	15,08 7,55	2,52 2,17	0,028

Legend:

MD: Ménière's Disease

FS: Firm Surface

* T-Student test Significance level α = 0.05

## DISCUSSÃO

In our evaluation of 30 patients with overt Ménière's disease in the intercritical period, hearing loss, tinnitus, ear fullness, vertigo and other types of dizziness, were reported by all the patients; highlighted are neurovegetative manifestations, hypersensitivity to sound and limitations in daily activities because of these symptoms. Sensorineural hearing loss and potential summation relation and an action potential higher than 30% upon electrocochleography were identified in all the cases. The use of the Brazilian version^15^ of the Dizziness Handicap Inventory^14^ showed a moderate loss in the quality of life of the patients 16 and the analogue dizziness scale indicated a moderate influence of symptoms^19^. Upon vestibulometry, in a little more than half of the cases there were signs of peripheral vestibular dysfunctions.

In our series, Balance Rehabilitation Unit (BRU^TM^) posturography showed that the stability limit area of the Ménière's disease group was similar to the control group, showing that our patients with Ménière's disease have the ability to move their body mass center and keep balance without changing the support base. Nonetheless, contrary to our findings, the stability limit was significantly different in comparison to the control group, assessment by another procedure and using other analysis parameters^20^. We did not find literature quotes on the stability limit in patients with Ménière's disease defined upon Balance Rehabilitation Unit (BRU^TM^) posturography.

Patients with Ménière's disease had oscillatory velocities significantly higher than those of the control group on a firm surface and downwards optokinetic stimulation and firm surface and horizontal visual-vestibular interaction. The values from the elliptical area were significantly higher than those from the control group on a firm surface with the eyes shut, on firm surface and left side optokinetic stimulation; firm surface and downwards optokinetic evaluation and firm surface and horizontal visual-vestibular interaction, and firm surface and vertical visual-vestibular interaction. These data show the involvement of static balance when there is visual deprivation and visual conflict by means of optokinetic stimuli and stimuli which are part of the visual and vestibular systems. We have not found literature quotes on the oscillatory velocity changes and that of the elliptical area in patients with Ménière's disease defined upon Balance Rehabilitation Unit (BRU^TM^) posturography.

Our findings with patients with Ménière's disease, defined upon Balance Rehabilitation Unit (BRU^TM^) posturography are of difficult quantitative comparison with those from other types of posturography, because of differences between evaluation parameters and procedures. Nonetheless, we can qualitatively compare the results, stressing that there were body balance changes in patients with Ménière's disease in some of the sensorial situations evaluated^8^, ^9^, ^10^, ^11^, ^12^, ^13^. We noticed differences in the performance of patients with Ménière's disease in the assessment of body balance in relation to the control group, especially under eyes shut and eyes shut on foam sensorial situations^9^, ^10^, ^11^, ^12^, ^13^. Upon Balance Rehabilitation Unit (BRU^TM^) posturography we observed significant differences between the groups as to the elliptical area only under eyes shut. These data show that our patients with Ménière's disease did not have a reduction in postural control under the sensorial situation in which the proprioceptive information was changed or in which the Balance Rehabilitation Unit (BRU^TM^) posturography was not sensitive enough to find changes in this situation.

In our series, the patients with Ménière's disease had a greater difficulty in maintaining body balance, shown by the change in elliptical area, when evaluated under the sensorial situations of perturbation of the visual system and interaction between the visual and vestibular systems. Changes in body balance under the sensorial situation in which the visual and vestibular systems were changed or excluded were also described by other authors^8^, ^9^,^21^. According to the results found under these sensorial situations and with the literature findings, we can think that the patients with Ménière's disease are more dependant on the visual input than the individuals without vestibular system changes^12^,^22^.

Our results indicated that Balance Rehabilitation Unit (BRU^TM^) posturography is a method which can help in the identification of the sensorial changes associated with the body balance of patients with Ménière's disease, since it assesses body balance using the stimuli from daily life. The information on oscillatory velocity values and that of the elliptical area on posturography under visual deprivation, somatosensory or vestibular changes can contribute in a relevant way to the programming and to the follow up of body balance disorders in patients with Ménière's disease.

## CONCLUSION

Body balance evaluation by means of the Balance Rehabilitation Unit (BRU^TM^) posturography enables the identification of oscillation velocity abnormalities and elliptical area in patients with Ménière's disease, in the conditions in which there was no vision by means of optokinetic stimuli and stimuli which integrate the visual and vestibular systems.
